# Predictors of Proclivity, Enjoyment, and Acceptance of Non-Consensual Intimate-Image Distribution Among Greek University Students

**DOI:** 10.3390/ejihpe15080150

**Published:** 2025-08-04

**Authors:** Constantinos M. Kokkinos, Theano-Athanasia Papioti, Ioanna Voulgaridou

**Affiliations:** 1 Department of Primary Education, School of Education Sciences, Democritus University of Thrace, N. Hili, GR 68131 Alexandroupolis, Greece; tpapioti@eled.duth.gr; 2 School of Humanities, Hellenic Open University, GR 26335 Patras, Greece; voulgaridou.ioanna@ac.eap.gr

**Keywords:** proclivity, non-consensual intimate-image distribution, enjoyment, acceptance, university students, demographic characteristics

## Abstract

Objectives: The present study investigated proclivity for non-consensual intimate-image distribution and its related dimensions—enjoyment and acceptance—in relation to key demographic and relational variables, including gender, age, sexual orientation, frequency of dating app use, and current romantic relationship status. Methods: A total of 1735 Greek university students (mean age = 22 years, standard deviation = 6.18; 35.2 percent male) participated in an anonymous online survey. Pearson correlation analyses and multiple linear regression models were conducted to examine the associations and predictive value of the demographic variables on proclivity, enjoyment, and acceptance of non-consensual intimate-image distribution. Results: Men, younger participants, and those who reported more frequent use of dating applications or websites demonstrated higher proclivity for non-consensual intimate-image distribution. Gender and frequency of dating app use were also significant predictors of enjoyment, with men and frequent users reporting greater enjoyment. Regarding acceptance, sexual orientation was the only significant predictor, with non-heterosexual individuals indicating higher levels of acceptance. Romantic relationship status did not significantly predict any of the three outcome variables. Conclusions: These findings highlight the significance of gender, age, sexual orientation, and dating app engagement in understanding the psychological and behavioral dimensions of non-consensual intimate-image distribution. The results support the need for targeted prevention efforts and further research into the contextual and psychosocial factors associated with this form of image-based abuse.

## 1. Introduction

Non-consensual intimate-image distribution (NCIID)—the unauthorized dissemination or threat of dissemination of explicit images without the subject’s consent—has emerged as a pervasive form of digital harm, affecting victims across social, cultural, and technological contexts. While often referred to as “revenge porn” or technology-facilitated sexual violence, the present study adopts the term NCIID to reflect recent legal and scholarly shifts toward more precise, non-blaming language (DeKeseredy & Schwartz, 2016; StopNCII.org, 2025). This terminology avoids conflating disparate behaviors and underscores the centrality of consent in defining the harm.

Although no consensus definition exists (Harper et al., 2023; McGlynn et al., 2017), most accounts describe on a broad conceptualization of NCIID as the non-consensual sharing or threatened sharing of private sexual material. Perpetrators range from former partners to acquaintances and strangers, and their motives span entertainment, control, power, and humiliation. These acts typically occur in digital contexts where the absence of face-to-face cues and immediate social sanctions can lower inhibitions and diminish perceptions of wrongdoing (Henry & Powell, 2018).

Recent meta-analyses and scoping reviews (Benítez-Hidalgo et al., 2024; Ehman & Gross, 2019; Henry & Beard, 2024; Parton & Rogers, 2025; Patel & Roesch, 2022; Walker & Sleath, 2017) position NCIID within broader frameworks of image-based sexual abuse and digital sexual violence. In parallel, a substantial empirical literature demonstrates that demographic attributes—such as gender (e.g., Karasavva & Forth, 2022; Zvi, 2022), age (Sparks et al., 2023), and sexual orientation (Karasavva et al., 2022; Shechory Bitton et al., 2024)—and behavioral factors like dating app use (Said & McNealey, 2023) are consistently associated with both NCIID perpetration and victimization, underscoring a notable perpetrator–victim overlap.

The widespread adoption of digital technology has normalized practices like sexting, typically a consensual and intimacy-enhancing behavior (Kokkinos & Krommida, 2022). However, this digital intimacy also carries risks, especially the potential for non-consensual dissemination of sexual content (Henry & Powell, 2018). Sexting participants often engage in private exchanges via dating apps or personal messaging, but once shared, the content can be redistributed in ways that escape accountability. These platforms, including anonymous forums, classrooms, and peer groups, create environments where NCIID can occur and be perceived as trivial or normative.

To better understand who is likely to engage in or approve of NCIID, this study examines five observable predictors: gender, age, sexual orientation, dating app use frequency, and romantic relationship status. These predictors represent two core domains linked to image-based abuse: (a) demographic positions that shape power dynamics, sexual norms, and moral judgments; and (b) relational and technological settings that afford motives and opportunities for sharing intimate content. Each has been associated with online sexual risk-taking, digital aggression, and attitudes toward consent.

Guided by social learning, aggression, and online-disinhibition perspectives, the study focuses on three psychological dimensions of NCIID: proclivity (one’s self-reported likelihood or hypothetical willingness to engage in such behavior), enjoyment (the positive affective response to the act), and acceptance (the degree of moral or normative approval of NCIID). These dimensions are used to examine the psychosocial profile of potential perpetrators and to inform prevention strategies.

Social Learning Theory (Bandura, 1977) specifically suggests that behaviors like NCIID can be learned and reinforced through observation, particularly in online spaces where image-sharing is normalized and rarely punished. Thus, dating app use and relationship status are examined as contextual variables that provide modeling opportunities. Supporting this, studies show that frequent dating app users are more likely to perpetrate and experience NCIID (e.g., Said & McNealey, 2023). Gender, age, and sexual orientation are included not as proxies for internal traits but due to their established links to NCIID perpetration and victimization (Henry & Powell, 2018; Sparks et al., 2023; Zvi, 2022).

Empirical evidence suggests that men report higher proclivity and enjoyment in relation to NCIID, while gender differences in acceptance remain inconsistent (Karasavva et al., 2022). Age also lacks a stable association with NCIID, whether based on self-reports or official data (Attrill-Smith et al., 2021; Davidson et al., 2019). Similarly, no systematic differences in proclivity, enjoyment, or acceptance have been consistently observed between heterosexual and non-heterosexual individuals (Karasavva et al., 2022). However, digital context remains a key factor: users who frequently engage with dating apps are exposed to more opportunities for intimate exchanges and may benefit from anonymity, which has been linked to elevated NCIID behavior (Choi et al., 2018; Henry & Powell, 2018). Romantic partners—particularly those with access to private images—also show higher proclivity (Sirianni & Vishwanath, 2016).

Despite the relevance of these predictors, they remain underexplored in the Greek context, where NCIID was only recently criminalized (in 2021), and where police recorded 30 complaints in 2022—a likely underestimation given the stigma surrounding disclosure. Cultural norms surrounding sexuality and shame, particularly in relation to female sexual agency, remain highly conservative in Greek society (Ioannou, 2024; Polyzoidou, 2024), potentially amplifying the harms and social fallout of NCIID victimization. Moreover, public discourse around image-based abuse in Greece has been shaped by a few high-profile cases involving celebrities and influencers, often sensationalized in the media rather than addressed through systemic education or prevention efforts. Focusing on Greek university students, an emerging adult population with high levels of digital engagement and sexual experimentation (Arnett, 2000; Kokkinos & Krommida, 2022), this study tests whether men and frequent dating app users report higher NCIID proclivity and enjoyment. No gender effect is expected for acceptance, and no a priori predictions are made for age. Effects of sexual orientation and relationship status are examined exploratorily. By mapping these demographic and digital behavioral variables onto proclivity, enjoyment, and acceptance, the study seeks to advance context-sensitive understanding and prevention of NCIID.

### 1.1. Gender Differences in Proclivity, Enjoyment, and Acceptance of NCIID

Research on gender and NCIID yields mixed patterns: several studies frame NCIID as a form of gender-based violence and report higher female victimization (Hall & Hearn, 2017; Bothamley & Tully, 2018), yet large-scale surveys also show comparable rates for men and women or even a higher proportion of male victims under certain conditions (Attrill-Smith et al., 2021; Henry et al., 2018; Powell et al., 2019). Methodological differences—official records versus anonymous surveys—and cultural factors such as gendered stigma around disclosure likely underlie these discrepancies, with women typically facing greater reputational harm and victim-blaming, thereby influencing reporting patterns (Powell & Henry, 2017; Henry & Beard, 2024).

Behaviorally, men are generally more engaged in online sexual activities and are over-represented among those who distribute explicit content (Liu & Zheng, 2020; Garcia et al., 2016), and relationship contexts that grant access to intimate images can further elevate men’s proclivity (Sirianni & Vishwanath, 2016). Several studies also indicate that men report significantly higher levels of proclivity, enjoyment, or moral disengagement related to NCIID and similar behaviors (Courtice et al., 2021; Powell & Henry, 2017; Ruvalcaba & Eaton, 2020). Against this backdrop, the present study predicts that men will report higher NCIID proclivity (H1a) and enjoyment (H1b) than women, while acceptance is expected to show no gender difference (H1c), thus accommodating the weight of contradictory findings while acknowledging gendered sociocultural dynamics.

### 1.2. Age-Related Variations in Proclivity, Enjoyment, and Acceptance of NCIID

Evidence on age-related patterns in NCIID is mixed and often reflects whether scholars measure proclivity (hypothetical willingness) or victimization (real-world incidents). Large-scale proclivity surveys detect no cohort that is disproportionately inclined to share images without consent (e.g., Attrill-Smith et al., 2021), yet other studies, particularly those assessing actual perpetration, have identified younger individuals as more likely to engage in or approve of such behaviors (e.g., Gámez-Guadix et al., 2022; Patchin & Hinduja, 2020; Valkenburg & Peter, 2011; Walker & Sleath, 2017). Victimization research also alternates between identifying young adults, typically 18- to 25-year-olds who sext frequently (CCRI, 2014; O’Connor et al., 2018), and somewhat older groups (26–33 or even 34–41 years) as the most affected (Davidson et al., 2019; Eaton et al., 2017).

This discrepancy likely stems from developmental timing: younger cohorts normalize sexting earlier, raising exposure risk, whereas perpetration requires both time to accumulate intimate material and the relational breakdowns that trigger misuse, conditions more common in later young adulthood. UK police reports from 2019 to 2022, for example, show the highest number of NCIID complaints in the 18–29 bracket, but a substantial secondary peak in the 30–39 bracket, illustrating a graduated rather than age-exclusive risk. Given these inconsistent trends, the present study predicts no meaningful age differences in NCIID proclivity (H2) and treats age effects on enjoyment and acceptance as exploratory, recognizing the weak and heterogeneous associations reported, to date.

### 1.3. Sexual Orientation as a Predictor of Proclivity, Enjoyment, and Acceptance of NCIID

Empirical work on sexual orientation and NCIID remains sparse and inconclusive. Sexting studies show higher participation among non-heterosexual adults in some samples (e.g., Galovan et al., 2018) but not others (Gordon-Messer et al., 2013), and these behavioral patterns cannot be assumed to translate into NCIID. The few investigations that address orientation directly paint a mixed picture: non-heterosexual men appear twice as likely as non-heterosexual women to distribute explicit images (Garcia et al., 2016), web-scrape analyses reveal offending across all orientations (Hall & Hearn, 2018), and survey data indicate comparable levels of proclivity among heterosexual and non-heterosexual university students (Karasavva et al., 2022).

However, several more recent studies have found higher rates of NCIID perpetration among non-heterosexual individuals, particularly in younger samples, suggesting that sexual orientation may be an important correlate of digital sexual aggression (e.g., Reuter et al., 2017). These differences may be interpreted through the minority stress framework (Meyer, 2003), which suggests that non-heterosexual individuals experience unique psychological burdens—including internalized stigma, peer rejection, and emotional dysregulation—that are associated with heightened interpersonal conflict and aggression (Edwards et al., 2015; Hatzenbuehler, 2009). Indeed, research on intimate partner violence (IPV) consistently finds elevated rates of perpetration among non-heterosexual youth and young adults compared to heterosexual peers (Martin-Storey, 2015; Mustanski et al., 2014), a pattern that may extend to digitally mediated forms of harm such as NCIID.

Nevertheless, findings regarding enjoyment and acceptance of NCIID remain more variable. For instance, Karasavva and Forth (2022) reported marginally higher enjoyment and uniformly high acceptance among non-heterosexual visitors to a revenge-porn site, the authenticity of “non-consensual” content on such platforms and the site-based sampling frame limit generalizability. Given this patchwork of results and the methodological caveats, the present study advances three orientation-related hypotheses: (H3a) sexual orientation will not differentiate NCIID proclivity; (H3b) orientation will likewise show no systematic association with NCIID enjoyment; and (H3c) non-heterosexual participants will report higher acceptance of NCIID than exclusively heterosexual participants.

### 1.4. Dating App Use as a Predictor of Proclivity, Enjoyment, and Acceptance of NCIID

Emerging social media and dating app technologies have reshaped courtship by expanding opportunities for contact while simultaneously widening avenues for sexual harm: perpetrators can exploit platform affordances—anonymity, dissociative identities, message deletion, geolocation data, and cross-linked accounts—to extend their reach and evade detection (Choi et al., 2018; Suler, 2005; Rowse et al., 2022; Phan et al., 2021). Although exchanges of personal information and intimate images within these apps are typically consent-based and context-specific (Nissenbaum, 2011), the same digital intimacy can render users vulnerable to NCIID when content is redistributed after relationships sour.

Usage patterns are demographically stratified: men constitute roughly 60% of the user base, uptake peaks between ages 18–24, and older adults often join following life transitions such as divorce or widowhood (Blackwell et al., 2015; Chin et al., 2019; Smith, 2016; Stephure et al., 2009). Building on these observations, we predict that younger adults (18–25) will report higher dating app use than those aged 26+ (H4a), that men will report heavier use than women (H4b), and that greater usage frequency will correlate with heightened NCIID proclivity (H4c) and enjoyment (H4d); the link between app use and NCIID acceptance is examined exploratorily (H4e).

### 1.5. Relationship Status and Its Association with Proclivity, Enjoyment, and Acceptance of NCIID

Stable romantic relationships, by virtue of the intimate self-disclosures they encourage, appear to create conditions under which NCIID can flourish; over half of recorded NCIID cases involve a current or former partner as the perpetrator (Karasavva & Forth, 2022; Phan et al., 2021; Sirianni & Vishwanath, 2016). Deeper emotional bonds and dependence accrued over time heighten the stakes of a breakup; feelings of betrayal—especially after infidelity—may catalyze retaliatory behaviors such as NCIID (Petruccelli et al., 2014). Both ongoing and dissolved partnerships can therefore facilitate the behavior: the former through power imbalances and coercion, the latter through revenge motives. On this basis we hypothesize that individuals with current or past long-term romantic relationships will report higher NCIID proclivity than those without such experience (H5), whereas associations between relationship status and the enjoyment or acceptance of NCIID remain exploratory given the paucity of prior evidence.

### 1.6. The Present Study

Drawing on Social Learning Theory to integrate modeling, person–situation dynamics, and digital anonymity, the present study examines how gender, age, sexual orientation, dating app use, and romantic relationship status predict proclivity, enjoyment, and acceptance of NCIID among Greek university students in emerging adulthood (18–25 years). This population is particularly relevant, as they exhibit high levels of digital intimacy and frequent engagement with platform-mediated romantic or sexual interactions (Kokkinos & Krommida, 2022). These interactions may heighten exposure to image-based content and to the digital affordances—such as anonymity, ephemerality, and ease of redistribution—that facilitate NCIID while reducing social accountability (Choi et al., 2018; Phan et al., 2021; Suler, 2005).

By linking these demographic and technological factors to psychological tendencies toward NCIID, the study addresses critical gaps in the literature. Moreover, it responds to recent calls for culturally contextualized investigations into image-based abuse in under-researched settings, such as Greece, where NCIID was only recently criminalized and empirical data remain limited. In doing so, the study aims to refine the psychosocial profile of potential perpetrators and inform prevention strategies tailored to digitally connected young adults.

## 2. Material and Methods

### 2.1. Participants and Procedure

Participants were recruited between December 2022 and March 2023 through Facebook posts in online student communities affiliated with Greek universities. The study was advertised as a voluntary, anonymous survey on digital behaviors and interpersonal relationships, and a reminder post was shared one month after the initial recruitment. Interested individuals accessed the survey via a secure Limesurvey link. Upon entering the platform, participants were presented with an informed consent form outlining the study’s purpose, their rights (including the right to withdraw at any time), and data confidentiality assurances. Only those who consented proceeded to the questionnaire.

A total of 1927 university students initially accessed the survey platform and submitted responses. To ensure data quality and reduce the possibility of bot or spam responses, the survey included an attention check item embedded midway through the questionnaire (e.g., “Please select ‘Strongly Agree’ for this item”). Responses that failed this attention check (*n* = 112) or showed signs of patterned or implausible response behavior, such as completing the full survey in less than 3 min were excluded from the final dataset (*n* = 80). IP checks were also used to prevent multiple entries from the same user (*n* = 0). After applying these exclusion criteria, the final analytic sample consisted of 1735 participants.

Participants ranged in age from 18 to 55 years (M = 22.00, SD = 6.18), with the majority (86.2%) falling in the 18–25 age group. This age band corresponds to the developmental period of emerging adulthood and was the primary population of interest in the present study. Given the skewed age distribution and consistent with digital behavior trends indicating peak engagement with social technologies during young adulthood, we categorized participants into two groups: 18–25 years (n = 1495; 86.2%) and 26+ years (*n* = 240; 13.8%). The older group was retained for exploratory comparison; however, it should be noted that participants over the age of 40 were very few, and findings primarily reflect the digital behaviors and attitudes of younger adult students.

In terms of sex assigned at birth, 611 participants (35.2%) identified as male and 1124 (64.8%) as female. Regarding sexual orientation, 281 participants (16.2%) identified as non-exclusively heterosexual (e.g., bisexual, pansexual, or other). In terms of romantic involvement, 765 participants (44.1%) reported being in a romantic relationship at the time of the survey. Additionally, 1020 participants (58.8%) reported having been in a romantic relationship at some point within the past year. These categories were not mutually exclusive; some participants indicated both current and past romantic relationships. To clarify, 41.2% of the sample (*n* = 715) were not in a romantic relationship at the time of participation, meaning they were single. Of these, some had been in a relationship earlier in the year, while others had not. The overlap between current and past relationship status accounts for the total percentages exceeding 100%.

The survey took approximately 15 min to complete. Participation was entirely voluntary, with no monetary or academic incentives provided. The study received approval from the host institution’s Ethics Committee. Portions of the dataset employed in the present study have appeared in a separate publication that investigated Dark Tetrad personality traits in relation to proclivity for NCIID (Kokkinos et al., 2025). That prior study analyzed only the proclivity outcome. The current article uses the same participant sample but addresses a distinct research question, examining demographic characteristics and dating app use as predictors and expanding the outcome space to include enjoyment and acceptance. Accordingly, none of the hypotheses, analyses, or results reported here overlap with those published previously.

### 2.2. Measures

**Demographics:** Two items were used to collect data on participants’ socio demographics gender and age.

**Non-Consensual Intimate-Image Distribution**: Proclivity toward NCIID was measured using the English language Revenge Porn Proclivity Scale (Pina et al., 2017), which has been translated and previously used in Greek university samples (Kokkinos et al., 2025). The scale includes six short vignettes depicting scenarios in which intimate images are shared without consent. Each vignette is followed by six to seven items assessing behavioral intent and emotional responses, rated on a five-point Likert scale (*1* = *Definitely would do the same*, to *5* = *Definitely would not do the same*).

The primary item in each vignette asked, “In this case, would you do the same?” Additional items assessed emotional and attitudinal reactions, such as excitement, control, enjoyment, anger, blame, and remorse (e.g., “How excited would you be?”; “To what extent would you blame the person who initially shared the photograph?”).

Proclivity scores were computed by summing responses to the behavioral intent item across the six vignettes (α = 0.76). Two additional subscales were computed: *NCIID enjoyment* (excitement, control, enjoyment; α = 0.87) and *NCIID approval* (anger, remorse, guilt; α = 0.80). The RPPS has demonstrated solid reliability and conceptual fit for operationalizing NCIID and was psychometrically validated for use in Greek samples.

**Sexual Orientation**: Sexual orientation was assessed using the 5-point Kinsey Scale (Kinsey et al., 1948), which captures self-reported patterns of sexual attraction on a continuum ranging from 1 (exclusively heterosexual) to 5 (exclusively homosexual). For analytic purposes, responses were grouped into two categories: exclusively heterosexual (score of 1) and non-exclusively heterosexual (scores of 2–5). This decision was driven by the need to ensure sufficient statistical power in subgroup analyses and is consistent with prior research that employed similar grouping strategies (e.g., Vrangalova & Savin-Williams, 2012).

**Dating App Usage:** Engagement with dating applications or websites was assessed through a two-step format. First, participants were asked: “Have you ever used a dating app or website (e.g., Tinder, Badoo, Grindr, etc.)?” (*0* = *No*, *1* = *Yes*). Those who responded “Yes” were then asked to indicate their frequency of use on a 5-point Likert scale, ranging from 1 (*Rarely or only once*) to 5 (*Very frequently*). This structure allowed us to differentiate between non-users and users while capturing meaningful variability among users. For the purposes of regression analyses, dating app use was treated as a continuous variable based on the frequency scale.

**Relationship Status:** Two items assessed participants’ relationship status (No = 0; Yes = 1): (a) “Are you in a romantic relationship now?”, (b) “Have you been in a romantic relationship within the last year?”. Participants could respond “yes” to both questions if, for example, they were in a current relationship that had lasted more than a year or if they had ended one relationship and entered a new one within the last 12 months. No definition of “romantic relationship” was provided in the questionnaire, allowing participants to interpret the term based on their personal understanding. Analyses involving relationship status treated both items separately to distinguish between current involvement and recent relationship history. The two variables were also combined to create a binary indicator of *any romantic involvement* for certain exploratory analyses.

### 2.3. Data Analysis Plan

To explore bivariate relationships among demographic variables and the outcome measures, Pearson correlation analyses were conducted. Specifically, correlations were calculated between age (treated as a continuous variable), gender (coded as 0 = female, 1 = male), sexual orientation (coded as 0 = exclusively heterosexual, 1 = non-heterosexual), dating app use (treated as a continuous frequency variable), relationship status (coded as *0* = *not currently in a romantic relationship*, *1* = *currently in a romantic relationship*), and the three outcome variables: proclivity, enjoyment, and acceptance of non-consensual intimate-image distribution.

To examine the unique predictive value of each demographic factor, a series of multiple linear regression analyses were performed. Three separate models were tested, each using one of the NCIID outcomes (proclivity, enjoyment, or acceptance) as the dependent variable. Age, gender, sexual orientation, dating app use frequency, and relationship status were simultaneously entered as predictors in each model. This approach enabled the assessment of each predictor’s contribution while statistically controlling for the others and avoided the risk of inflated Type I error associated with multiple group comparisons or multivariate testing. To adjust for multiple comparisons across the three regression models, a Bonferroni correction was applied, setting the threshold for statistical significance at *p* < 0.017 (i.e., 0.05/3). Predictors with *p*-values between 0.017 and 0.05 were interpreted as marginally significant.

Regression assumptions were tested and satisfied, and collinearity diagnostics were conducted to ensure the robustness of the models. All tolerance values exceeded 0.90 and VIF values were below 1.2, indicating low multicollinearity. Residuals were normally distributed and showed homoscedasticity. All analyses were conducted using IBM SPSS Statistics (Version 26).

## 3. Results

Bivariate Pearson correlations were calculated to examine associations among demographic variables and the three NCIID outcomes: proclivity, enjoyment, and acceptance. As shown in Table 1, gender was significantly associated with both NCIID proclivity (*r* = −0.13, *p* < 0.01) and enjoyment (*r* = −0.16, *p* < 0.01), indicating higher levels among men. Dating app use, treated as a continuous variable reflecting frequency of use, was significantly associated with greater proclivity (*r* = −0.18, *p* < 0.01), enjoyment (*r* = −0.15, *p* < 0.01), and acceptance (*r* = 0.07, *p* < 0.01). Sexual orientation was negatively correlated with NCIID acceptance (*r* = −0.10, *p* < 0.01), indicating higher acceptance among non-heterosexual individuals. Age was weakly negatively correlated with proclivity (*r* = −0.05, *p* < 0.05), and relationship status was positively correlated with proclivity (*r* = 0.06, *p* < 0.05), suggesting that younger participants and those not in a relationship may report higher proclivity toward NCIID.

To further examine the relative contribution of demographic predictors to NCIID outcomes, three multiple linear regressions were conducted (Table 2). The overall model for NCIID proclivity was statistically significant, *F*(5, 1725) = 27.91, *p* < 0.001, and explained 7.5% of the variance in proclivity (*R^2^* = 0.075, adjusted *R^2^* = 0.072, f^2^ = 0.081). Significant predictors included gender (β = −0.104, *p* < 0.001) and dating app use frequency (β = 0.236, *p* < 0.001), both of which remained statistically significant after Bonferroni correction. Age (β = −0.057, *p* = 0.016) was marginally significant under the corrected threshold (*p* < 0.017). These results suggest that men, younger individuals, and more frequent dating app users reported higher proclivity toward NCIID. Sexual orientation and relationship status were not significant predictors.

The regression model for NCIID enjoyment was also significant, *F*(5, 1717) = 25.11, *p* < 0.001, accounting for 6.8% of the variance (*R^2^* = 0.068, adjusted *R^2^* = 0.065, f^2^ = 0.073). Gender (β = −0.138, *p* < 0.001), and dating app use frequency (β = 0.207, *p* < 0.001) remained significant under the Bonferroni-corrected threshold. Age (β = −0.047, *p* = 0.050) did not reach significance. Men and more frequent dating app users reported greater enjoyment of NCIID scenarios. Sexual orientation and relationship status were not significant.

The model predicting NCIID acceptance was also statistically significant, albeit with a small effect size, *F*(5, 1717) = 5.04, *p* < 0.001, explaining 1.4% of the variance (*R^2^* = 0.014, adjusted *R^2^* = 0.012, f^2^ = 0.014). Only sexual orientation significantly predicted acceptance (β = −0.089, *p* < 0.001) and remained significant after correction with non-heterosexual participants showing greater acceptance of NCIID. Age (β = 0.044, *p* = 0.072) and dating app use frequency (β = −0.046, *p* = 0.060) showed marginal effects. Gender and relationship status were not significant.

## 4. Discussion

The present study aimed to investigate proclivity toward NCIID among Greek university students, along with related attitudes such as acceptance and enjoyment, focusing on demographic predictors such as gender, age, sexual orientation, dating app usage, and relationship status. Although NCIID has received some scholarly attention in Greece, existing studies are limited in scope, particularly concerning psychological proclivity and behavioral predictors in emerging adulthood. Given the increasing prevalence of digital intimacy among university students and the recent legal recognition of NCIID in the Greek penal code, there is a pressing need to understand how individual and contextual factors contribute to this form of image-based abuse. Unlike countries such as the U.S., U.K., or Australia, where NCIID research has proliferated in tandem with robust legislative frameworks and prevention programs, Greece lacks widespread educational campaigns, institutional support structures, and public awareness around digital sexual harm. Furthermore, societal norms around modesty and gender roles and the stigma attached to online sexual behavior, particularly for women, may compound both the prevalence and the psychological impact of NCIID. By targeting a high-risk yet understudied population, this study expands upon international findings and provides culturally relevant insights that can inform targeted prevention efforts and policy development. It also highlights the importance of context-sensitive interventions that address not only individual-level predictors but also the broader sociocultural dynamics that shape responses to image-based abuse in Greece.

Consistent with H1a, the results revealed significant gender differences in NCIID proclivity, with men scoring higher than women. This aligns with international findings suggesting that men are more likely to perpetrate or endorse NCIID (Hall & Hearn, 2017; Karasavva & Forth, 2022; Powell et al., 2019) and is further supported by recent scholarship situating NCIID within broader patterns of digital sexual violence and gendered harm (Benítez-Hidalgo et al., 2024; Henry & Beard, 2024; Parton & Rogers, 2025). Although some studies (e.g., Powell et al., 2019) have found similar rates of victimization among men and women, others indicate that women are disproportionately targeted and harmed. In Greece, data from the Department of Electronic Crime Prosecution suggest that women comprise the majority of reported victims. These trends reflect broader dynamics of gender-based violence, with NCIID often used as a tool of control, humiliation, and punishment by male perpetrators (McGlynn et al., 2017; Henry & Powell, 2016).

Gámez-Guadix et al. (2022) further suggest that men’s higher engagement in provocative online behavior may be a contributing factor. From a social learning perspective, higher male proclivity may reflect gender-differentiated modeling in online peer groups where NCIID is normalized and rarely sanctioned, reinforcing expectancies that such behavior is both acceptable and rewarding.

Regarding NCIID enjoyment (H1b), men reported significantly greater enjoyment than women, replicating earlier findings by Karasavva and Forth (2022). This gender gap may reflect enduring sexual double standards that normalize men’s sexual agency while harshly judging women for similar behaviors (Hearn & Hall, 2019). Recent work also highlights the persistence of these gender norms in shaping digital sexual behaviors and responses to violations (Henry & Beard, 2024). However, when it came to the acceptance of NCIID (H1c), no significant gender differences were found. This pattern supports the results of Karasavva and Forth (2022) and may be attributed to the normalization of victim-blaming attitudes, such as perceiving victims as promiscuous or downplaying the severity of image-based violations (Harper et al., 2023). These cognitive distortions may foster a general tolerance for NCIID across gender groups. From the perspective of Social Learning Theory, these attitudes may be socially reinforced in online communities, where morally questionable behaviors receive social validation and lack consequences.

In relation to age (H2), the results indicated no significant differences in NCIID proclivity between younger (18–25) and older (26+) participants. This finding is consistent with prior research reporting similarly weak or non-significant age effects in NCIID contexts (Attrill-Smith et al., 2021; Davidson et al., 2019; Henry et al., 2018; O’Connor et al., 2018) and aligns with more recent empirical work that fails to identify strong or systematic age patterns in NCIID behavior (Sparks et al., 2023). One plausible explanation is that digital sexual behaviors—such as sexting—have become normalized across age cohorts, increasing general exposure to intimate content without necessarily increasing proclivity for non-consensual distribution (Kokkinos & Krommida, 2022; Stephure et al., 2009). Similarly, no significant age differences were found in NCIID enjoyment or acceptance, further suggesting that perceptions of and responses to such behaviors may not be strongly age-dependent among adults.

This pattern may reflect a broader societal shift in which digital sexual expression has become desensitized through repeated exposure. As noted in recent reviews (Parton & Rogers, 2025), the widespread availability of explicit content online may reduce the perceived moral weight of sharing such material—particularly in hypothetical scenarios. Additionally, prior research has shown that frequent exposure to aggressive or explicit content in online spaces—such as memes, videos, or forums—can diminish emotional sensitivity and promote increased tolerance for digital harm (Anderson & Bushman, 2018; Runions et al., 2013). Based on Social Learning Theory, when a behavior becomes culturally widespread through ubiquitous sexting, age differences are narrow because observational learning opportunities saturate across cohorts. The Online Disinhibition Effect may also play a role, as digital environments characterized by anonymity and invisibility reduce the salience of age-related social norms that would otherwise moderate behavior.

On the contrary, regarding Hypothesis H3a, the results indicated no significant difference in NCIID proclivity between exclusively heterosexual and non-heterosexual participants. This finding aligns with several previous international studies (Hall & Hearn, 2018; Karasavva & Forth, 2022) and is further echoed in newer evidence showing mixed or minimal differences in NCIID tendencies across sexual orientations (Shechory Bitton et al., 2024). It may be explained by the fact that both heterosexual and non-heterosexual individuals engage in digital intimacy practices such as sharing sensitive personal content with partners (Gordon-Messer et al., 2013).

Regarding enjoyment and acceptance, Hypothesis H3b—which proposed no difference in enjoyment—was supported, as both groups reported comparable levels of enjoyment in NCIID scenarios. However, Hypothesis H3c, which expected no difference in acceptance, was not supported. Specifically, non-heterosexual participants reported significantly greater acceptance of NCIID compared to their heterosexual counterparts. It is important to note, however, that “acceptance” in this study does not necessarily reflect moral approval. Rather, it may encompass a broader attitudinal orientation that includes elements of normalization, desensitization, or even resigned tolerance. For example, the higher acceptance reported by non-heterosexual participants may reflect greater exposure to online risk or a perceived inevitability of digital boundary violations, rather than genuine support for such behaviors. This interpretation aligns with broader research on minority stress and digital vulnerability, which suggests that marginalized individuals may develop more pragmatic or fatalistic attitudes toward online harms due to repeated exposure (Henry & Powell, 2018; Ruvalcaba & Eaton, 2020). Future research should aim to disentangle these overlapping constructs by employing more granular or multidimensional measures of response to NCIID.

These findings partially align with the results of Karasavva et al. (2022), who found high levels of enjoyment and acceptance in their undergraduate sample. What makes these findings striking is that such permissive attitudes were observed in a normative sample (primarily cisgender, heterosexual women), suggesting that enjoyment and acceptance of NCIID are not limited to marginalized or high-risk groups. The current findings suggest that while the affective response to NCIID (i.e., enjoyment) may be similar across sexual orientations, broader attitudes toward the acceptability of this behavior may diverge—possibly reflecting differences in socialization, community norms, or prior exposure to online risks. From an Online Disinhibition Effect perspective, the reduced accountability and depersonalization of online interactions may differentially affect subgroups based on prior online experiences, shaping attitudes toward the moral permissibility of NCIID.

Hypothesis H4b was unsupported, as women, not men, reported slightly higher usage—an outcome that may signal shifting gender norms in which women increasingly initiate online romantic and sexual interactions (Hobbs et al., 2017) and may also reflect the female-skewed composition of the present sample. In line with H4c, frequent users of dating apps were more likely to express higher proclivity toward NCIID. This relationship may be attributed to the increased exposure to sexualized interactions, norm violations, or depersonalization dynamics that dating platforms can facilitate (Choi et al., 2018; Phan et al., 2021). This is in line with more recent work highlighting how dating apps increase exposure to sexual risks and normalize risky behaviors (Said & McNealey, 2023). Digital intimacy may blur boundaries or desensitize individuals to the ethical implications of non-consensual sharing. The Online Disinhibition Effect offers a compelling explanation here, suggesting that anonymity and ephemerality within dating platforms lower psychological barriers and foster impulsivity, increasing the likelihood of deviant digital behaviors.

Support for H4d was mixed. Participants who used dating apps more frequently reported greater enjoyment of NCIID scenarios, potentially reflecting heightened arousal or curiosity shaped by online sexual cultures. However, these same individuals reported lower levels of acceptance of NCIID. This divergence suggests a complex interplay: while digital dating experiences may increase emotional reactivity to NCIID, they might simultaneously foster greater moral awareness or fear of being victimized, reducing explicit approval of such behaviors. Moreover, the transitory and anonymous affordances of dating apps may erode self-regulation, thereby heightening both the willingness and the thrill associated with redistributing intimate images. Paradoxically, the same disinhibitory context may also heighten awareness of victimization risk, explaining why frequent users reported lower moral approval—an ambivalence predicted by the ‘toxic simultaneity’ of disinhibition and risk appraisal in Suler’s model.

In line with hypothesis H5, it was anticipated that individuals currently or previously involved in a stable romantic relationship would demonstrate greater proclivity toward NCIID, given the increased likelihood of sharing intimate content within such partnerships. Prior research has shown that romantic partners are more likely to exchange sensitive materials such as explicit messages or images compared to those in casual or online-only relationships (Phan et al., 2021; Sirianni & Vishwanath, 2016). This dynamic, combined with emotional vulnerability following breakups, may elevate the risk of NCIID perpetration as a form of retaliation—particularly in cases involving betrayal or unresolved conflict (Sirianni & Vishwanath, 2016). Supporting this, Karasavva and Forth (2022) found that ex-partners were frequently identified as NCIID perpetrators. From a social learning perspective, the romantic relationship context may provide both the source of sensitive material and the behavioral modeling (e.g., revenge narratives) that normalize retaliatory digital actions.

Despite this theoretical foundation, the present study did not find a significant difference in proclivity between participants in romantic relationships and those who were not. This unexpected result may reflect a growing normalization of intimate image-sharing within romantic contexts, where individuals may implicitly accept certain risks or view such behaviors as part of modern relationship dynamics (Knight, 2011). Moreover, the absence of significant findings regarding NCIID enjoyment and acceptance further complicates the expected role of relational status. These attitudinal and affective responses may be shaped less by relationship involvement per se and more by other individual-level or behavioral factors—such as gender, sexual orientation, or digital platform usage.

It is also possible that as sociocultural understandings of privacy, consent, and sexual agency continue to evolve in digital spaces, the relevance of relationship status as a predictor is diminishing (Henry & Beard, 2024). Future studies should consider more nuanced relational variables—such as relationship quality, satisfaction, or patterns of communication and trust—to better capture the complexities underlying NCIID proclivity and attitudes. Finally, the absence of a relationship status effect may reflect the influence of situational affordances (e.g., platform design, anonymity features) that shape behavior irrespective of relational context.

These findings should be interpreted in light of several methodological constraints. Although statistically significant, the proportion of variance explained by the models was modest (f^2^ = 0.014−0.081), indicating that other psychosocial or dispositional variables likely contribute to NCIID behaviors beyond demographics. First, reliance on a Greek university convenience sample recruited via snowballing limits generalizability; future work should draw on more heterogeneous age, cultural, and non-student populations. A pronounced age skew—only 13.8% of participants were ≥26 years—and dichotomous grouping (18–25 vs. 26 +) reduces statistical power for developmental comparisons, while a substantial gender imbalance (female ≈ 2 × male) correspondingly narrows confidence in sex-linked conclusions.

Sexual orientation was collapsed into “exclusively heterosexual” versus “non-exclusively heterosexual” to retain cell sizes, masking potential differences among bisexual, mostly heterosexual, and exclusively homosexual respondents. Although the Kinsey Scale captures sexual attraction, it does not measure sexual identity or behavior, which may have introduced mismatches between reported orientation and lived experience. No additional self-report data were collected on identity labels (e.g., bisexual, gay, queer), and our binary classification may have obscured meaningful within-group differences. To address these limitations, future research should prioritize the collection of finer-grained sexual orientation data, including both dimensional measures (e.g., Kinsey-type scales) and categorical identity labels (e.g., bisexual, gay, queer), in order to more accurately reflect the full spectrum of orientations and capture within-group variability. Additionally, although participants were asked about their gender, no data on gender identity beyond binary male/female categories were collected, limiting the ability to assess or report gender diversity.

Exclusive use of self-report measures raises the possibility of social desirability under-reporting, suggesting the value in complementary qualitative or behavioral methods. Finally, persistent cross-study variability in definitions, sampling frames, and instruments hampers prevalence estimates and comparability; progress will require convergence on standardized operationalization and validated tools for non-consensual intimate-image distribution. Finally, this study focused on the main effects of demographic variables on NCIID outcomes. Future research could expand this work by examining potential interactions among predictors—such as gender by dating app use or age by sexual orientation—particularly when supported by diverse samples that permit greater subgroup analysis.

Despite these limitations, the study makes important contributions to understanding NCIID proclivity, attitudes, and associated demographic and technological factors in emerging adults. By focusing on a culturally specific context and applying a social learning framework, this study enhances our understanding of individual susceptibility to NCIID behaviors while offering a foundation for future prevention and intervention efforts.

The findings also have significant practical implications. University students, as digital natives, are particularly vulnerable to online sexual exploitation due to the widespread use of sexting and dating apps during emerging adulthood—a critical period of identity formation (Arnett, 2000). The study highlights the urgent need for educational interventions that promote digital ethics and responsible online behavior. Universities should consider implementing student-centered programs on online sexual well-being that emphasize safety, consent, and the psychological impact of NCIID. Such interventions should equip students with skills to navigate risky online sexual behaviors, manage coercive situations, and understand the broader social consequences of non-consensual image sharing.

Dissemination of these findings through university counseling centers, student newsletters, or awareness campaigns hosted on institutional websites could raise awareness and encourage help-seeking behaviors. Moreover, early interventions—targeted at adolescents prior to university—may be especially effective. Programs that foster empathy, critical thinking, and digital responsibility during this formative stage could mitigate the emergence of harmful behaviors such as NCIID in later years.

In summary, while the study has limitations related to sample representativeness, measurement scope, and reliance on self-report, it remains an important step toward understanding the psychological and contextual factors associated with NCIID. By focusing on proclivity, enjoyment, and acceptance, and examining their associations with key demographic and behavioral predictors, the study advances theoretical discourse and offers actionable insights for prevention. Future research should build on these findings using more diverse samples and refined methodologies to develop targeted interventions that can reduce the prevalence and impact of NCIID.

## Figures and Tables

**Table 1:** Intercorrelations among variables (N = 1727).

Variable	1	2	3	4	5	6	7
1. Age ^1^							
2. Gender ^2^	−0.07 **						
3. Sexual Orientation ^3^	−0.07 **	0.08 **					
4. Dating App Use ^4^	0.00	−0.09 **	−0.15 **				
5. Relationship Status ^5^	−0.17 **	−0.06 *	0.07 **	−0.02			
6. NCIID Proclivity	−0.05 *	−0.13 **	0.00	−0.18 **	0.06 *		
7. NCIID Enjoyment	−0.03	−0.16 **	−0.01	−0.15 **	0.02	0.63 **	
8. NCIID Acceptance	0.05	0.03	−0.10 **	0.07 **	−0.01	−0.08 **	−0.04

*Note*. Pearson correlations are reported. ^1^ Age is measured as a continuous variable in years, ^2^ Gender coded as 0 = female, 1 = male. ^3^ Sexual orientation coded as 1 = exclusively heterosexual, 2 = non-heterosexual. ^4^ Dating app use reflects the frequency of use and is treated as a continuous variable. ^5^ Relationship status coded as 0 = not currently in a romantic relationship, 1 = in a romantic relationship. * *p* < 0.05, ** *p* < 0.01.

**Table 2:** Summary of multiple regression results with effect sizes.

Predictor	NCIID Proclivity				NCIID Enjoyment				NCIID Acceptance			
	β	*p*	f^2^	95% CI	β	*p*	f^2^	[−0.094, 0.000]	β	*p*	f^2^	95% CI
Age	−0.057	0.016	0.01	[−0.103, 0.011]	−0.047	0.050	0.008	[−0.181, 0.095]	0.044	0.072	0.004	[−0.004, 0.092]
Gender	−0.104	<0.001	0.02	[−0.148, 0.060]	−0.138	<0.001	0.03	[−0.086, 0.008]	0.039	0.109	0.003	[−0.008, 0.086]
Sexual Orientation	−0.038	0.111	0.005	[−0.085, 0.009]	−0.039	0.104	0.005	[0.159, 0.255]	−0.089	<0.001	0.02	[−0.131, 0.047]
Dating App Use	0.236	<0.001	0.06	[0.188, 0.284]	0.207	<0.001	0.05	[−0.046, 0.044]	−0.046	0.060	0.006	[−0.094, 0.002]
Relationship Status	0.029	0.215	0.002	[−0.017, 0.075]	−0.001	0.979	0.0	[−0.094, 0.000]	0.006	0.810	0.0	[−0.042, 0.054]

## Data Availability

The data presented in this study are available on request from the corresponding author due to ethical concerns about confidentiality.
