# Correction: Kokkinos et al. (2025). Predictors of Proclivity, Enjoyment, and Acceptance of Non-Consensual Intimate-Image Distribution Among Greek University Students. *European Journal of Investigation in Health, Psychology and Education*, *15*(8), 150

**DOI:** 10.3390/ejihpe15100215

**Published:** 2025-10-21

**Authors:** Constantinos M. Kokkinos, Theano-Athanasia Papioti, Ioanna Voulgaridou

**Affiliations:** 1 Department of Primary Education, School of Education Sciences, Democritus University of Thrace, N. Hili, GR 68131 Alexandroupolis, Greece; 2 School of Humanities, Hellenic Open University, GR 26335 Patras, Greece

## Text Correction

The authors wish to make the following corrections to their paper (Kokkinos et al., 2025). There is an error in the ninth paragraph of the Section 4 Discussion (first three sentences). The author stated:

“These findings partially align with the results of Karasavva and Forth (2022) who found high levels of enjoyment and acceptance in both groups. However, their sample consisted of users from a revenge porn website, which may have inflated permissive attitudes toward such content. As a result, some caution is warranted when interpreting elevated acceptance rates.”

This statement contains the following two inaccuracies:

1. The findings on enjoyment and acceptance derive from Karasavva, V., Swanek, J., Smodis, A., & Forth, A. (2022). From myth to reality: Sexual image abuse myth acceptance, the Dark Tetrad, and non-consensual intimate image dissemination proclivity. Journal of Sexual Aggression, 29(1), 51–67, not from Karasavva & Forth (2022).
2. The sample in Karasavva et al. (2022) consisted of undergraduate students from a Canadian university, not users of a revenge porn website.

The corrected statement should replace the existing three sentences with:

“These findings partially align with the results of Karasavva et al. (2022), who found high levels of enjoyment and acceptance in their undergraduate sample. What makes these findings striking is that such permissive attitudes were observed in a normative sample (primarily cisgender, heterosexual women), suggesting that enjoyment and acceptance of NCIID are not limited to marginalized or high-risk groups.”

The authors state that the scientific conclusions are unaffected. This correction was approved by the Academic Editor. The original publication has also been updated.
